# My husband is not ill; he has memory loss - caregivers´ perspectives on health care services for persons with dementia

**DOI:** 10.1186/s12877-019-1090-6

**Published:** 2019-03-06

**Authors:** Randi Granbo, Elisabeth Boulton, Ingvild Saltvedt, Jorunn L. Helbostad, Kristin Taraldsen

**Affiliations:** 1 Department of Neuromedicine and Movement Science, Faculty of Medicine and Health Science, NTNU, 7491 Trondheim, Norway; 2 School of Health Sciences, Faculty of Biology, Medicine and Health, University of Manchester, Oxford Road, Manchester, M13 9PL UK; 3 Department of Geriatrics, St Olav University Hospital, Medisinsk klinikk, Avdeling for geriatri, 7030 Trondheim, Norway

**Keywords:** Caregiver, Dementia, Activity, Thematic analysis, Caregiver burden

## Abstract

**Background:**

To explore informal caregivers’ perspectives and perceived needs related to health care services/activities for older adults with dementia, in order to understand barriers and facilitators to participation. The study represents a first step, and explores challenges to overcome, in order to design new activities and services adapted to older adults with dementia.

**Methods:**

We used a qualitative approach where eight caregivers of people with a dementia diagnosis were included. We recruited participants from a counselling service centre, for home dwelling people with dementia and their families, in a Norwegian municipality. We transcribed data from two focus group interviews and completed analyses by use of Systematic Text Condensation method.

**Results:**

The findings indicate that current health care services for people with dementia do not meet the needs of either the people with dementia or their caregivers. The few activities/services offered are characterised by passivity and lack of individual and personalised care. Existing health care services and new activities should consider each individual’s resources, interests, and physical function to ensure that both people with dementia and their caregivers want to accept support.

**Conclusions:**

To develop health care services and activities for people with dementia, participation and involvement from both people with dementia and their caregivers is necessary. People with dementia are more than their diagnosis. Future health care providers have to widen their focus and consider the individual person with dementia.

## Background

In Europe, one in every 20 people over the age of 65 years has Alzheimer Disease (AD), the most common form of dementia, and between 50 and 80% are cared for at home. As the number of older adults in the population increases, so will the number of people living with dementia. If we include family members who are informal, unpaid caregivers for this group, the number of people affected by the disease doubles [1].

Informal caregivers provide the majority of care, and may postpone the need for formal long-term care for people with dementia. Being the primary caregiver to a person with dementia can put them at risk of health problems, with family members often describing a situation of stress and frustration. The term ‘caregiver burden’ is often used to encompass the physical, psycological, emotional, social and finacial stressors assosiated with the caregiving role [2].

Today policy makers in most countries encorage older adults to age at home, with unintentional negative consequences for family carers who care for people with dementia as a result [3]. The caregiver burden seem to be worsened with additional expectations to engage in self-care. Health care systems that depends on informal caregivers have to re-orientate toward the caregivers and acknowledge their needs for support [3].

Adequate support involves both reducing caregiver burden and improving quality of life for both caregivers and the family member with dementia. A multinational review of interventions to reduce dementia-caregiver burden found that most of these interventions have been unsuccessful [4]. Educational interventions and/or support can, however, reduce caregiver burden, especially if the support is given at the onset of care [5].

When a person’s cognition starts to decline, caregivers often hesitate to seek help or support from health care services, partly because they are unsure about the difference between normal ageing and early signs of dementia, or because they consider these signs of deterioating cognition as not serious or permanent. Fear of compounding myths, stereotypes and fears about dementia from the public are also reasons why some hesitate to ask for help [6].

In Norway, half of the community-dwelling persons with dementia receive home care. Twenty percent of them are users of day care centers, with 1.5 to 2 days spent on average per week. The amount of health care stays relatively constant over time in this group until nursing home admission. The last month before admission to a nursing home, persons with dementia in Norway receive only four hours of home care per week. In contrast, the amount of informal help is estimated to be around 160 h per month [7, 8].

Ninty percent of persons with dementia in Norway, receive help from informal caregivers. Decline in activities of daily living, neuropsychiatric symptoms, and living alone are factors associated with increased use of health services. Patients with dementia admitted to nursing homes show better physical health as compared with patients without dementia, indicating admission due to their dementia and not for physical health problems [7, 8].

Healthcare professionals have a responsibility to recognise caregiver burden [9] and to tailor interventions to individual circumstances and contexts, where caregiver burden occurs [9, 10]. Dementia specific and team-based approaches seem to be appropriate to support informal caregivers in the help-seeking process [11] and caregivers should receive appropriate, individual offers for both support and for respite [12].

There is a need for qualitative studies exploring the perceived needs of informal caregivers, and to examine the underlying relationships between different caregiver needs, to inform the development of effective interventions and practice of care professionals [13]. First-hand information and needs of informal caregivers will be of great importance to make sure that the delivery of support from health care services are adequate and in accordance with their needs [14].

The aim of this paper is thus to describe informal caregivers’ perspectives and perceived needs for health care services for older people with dementia in a municipality. The study is part of a collaborative project between researchers and clinicians in primary health care, which aims to develop new services for people with dementia.

## Methods

### Design

This explorative and qualitative study took place within primary health care in the Municipality of Trondheim in Norway. To gain more knowledge about the caregivers` perspectives and perceived needs for health care services for older people with dementia, focus groups were undertaken.

### Ethics

The Medical Ethics Committee approved the study (2014/1689/REK sør-øst A).

### Recruitment and participants

We included a purposive sample [15] of informal family caregivers from a counselling centre for people with dementia and their families. Participants have been caregivers for a prolonged period of time and have all attended an educational lesson for caregivers, and most of them participated regularly in conversational groups. They were informal caregivers for home-dwelling adults with dementia above 65 years of age, living in Trondheim, interested in talking about their experiences with health care services offered for older adults with dementia, and their perspectives about needs for services. We aimed at including family (both partners and offspring) caregivers and carry out two focus group interviews with 4–6 participants each [16]. This number means we could ensure that each participant had the opportunity and time to share experiences from being caregivers and we could collect in depth data.

We included eight participants in total their age ranged from 63 to 83 years (two daughters and six spouses). The first focus group included three women and two male spouses, the second group consisted of three women (one spouse and two daughters). To gain a greater understanding of the participants’ situation, we collected some data on caregiver burden where Table 1 presents caregiver burden scores from the Hospital Anxiety and Depression Scale (HADS) [17, 18] and the Relatives’ Stress Scale (RSS) [19]. Five participants had HADS scores higher than 15, indicating a severe disorder [17, 18]. Results on caregivers’ distress show that seven out of eight of the participants were in a high-risk group for psychiatric morbidity, with scores above 30 [20].

**Table 1:** Caregiver burden reported by the participants (*n* = 8)

Participant (relation and age)	D62	D63	FS69	MS75	FS82a	FS82b	MS81	FS82c
Anxiety scores (0–21)	15	11	15	10	5	–	12	13
Depression scores (0–21)	3	12	9	5	8	–	13	9
HADS Anxiety and Depression (0–42)	18	23	24	15	13	–	25	22
Caregiver distress (0–60)	36	38	36	39	34	45	19	33

*D* Daughter, *FS* Female Spouse (three of the FS at the same age is marked with a, b and c); *MS* Male Spouse; including caregivers’ age in years

### Data collection

We used focus groups to explore experiences, opinions, and perspectives of the individual participants [21]. Participants were free to share experiences and opinions on one or more topics [22]. Interaction between people, all having experiences as caregivers of a person with dementia, was essential to reveal information of importance to this study and was the main reason for choosing focus groups instead individual interviews. Focus groups provide the opportunity for participants to listen to others and refine their own ideas through discussion [23]. To ensure that the focus groups remained on topic, a moderator facilitated the interaction between participants [22].

We collected data through two separate focus groups, in December 2015 (*n* = 3) and March 2016 (*n* = 5). In total, eight caregivers of people with dementia participated: two daughters, four wives, and two husbands. Participants’ quotes are marked as D = Daughter; FS=Female Spouse; MS = Male Spouse, including caregivers’ age in years.

The first author moderated both focus groups and the last author was present at both events. The focus groups lasted 75 to 100 min each and participants gave consent for both focus groups to be audio recorded.

We developed a semi-structured interview guide with four themes and a final open question. The themes were: 1) Motivation for joining the focus group; 2) Perspectives of current health care services; 3) Knowledge of what services the municipality offers to people with dementia; 4) The utility of participation in activities outside the home for people with dementia. Finally, we invited the participants to discuss what services they would prefer for their mothers, husbands or wives.

### Data analysis

The last author transcribed the audiotaped material verbatim. In the analysis, we applied Malterud’s Systematic Text Condensation method, which is inspired by Giorgi’s phenomenological approach [24]. Three authors read the transcripts independently, in order to get a general sense of the total material and to identify themes, before we discussed and redefined the themes. After discussing the overall impression, the first author identified meaning units related to the themes. We discussed the coding in meetings between the co-authors. Finally, two of the authors presented the main themes and codes for a sample of caregivers [25, 26], before the final themes were set. Present at this meeting were informants (*n* = 4) from the focus groups and caregivers (*n* = 3) from the same conversational group for caregivers that wanted to take part although they did not have time for joining the focus group interviews. All co-authors approved the final analysis. The first author selected illustrative quotes for the themes and codes.

## Results

Participants’ most explicit reason for taking part in this study was to contribute to a better understanding of needs, in order to improve health care services for home-dwelling people with dementia and their caregivers in the future. Three themes emerged from the analysis: 1) A gap between current health care services and perceived needs; 2) Caregivers’ role is all-consuming; 3) Involvement and participation is necessary for improving services. Table 2 shows the themes and meaning units.

**Table 2:** Themes and meaning units

Themes	Meaning Units	
A gap between current health care services and perceived needs	Code 1	Passive care
	Code 2	Lack of personalisation
Caregivers’ role is all-consuming	Code 3	The sole caregiver
	Code 4	Loss of social network
	Code 5	Conflicting desires
Involvement and participation is necessary to improve services	Code 6	Person centred services
	Code 7	Individualised support

### A gap between current health care services and perceived need

All participants had experiences with one or more services offered by the Municipality. A common experience was described as passive care and a lack of individualisation. Those with experiences with home care services reported a service characterised by unpredictability, with too many employees and unstable staff teams.

### Passive care

The most common service offered was group-based, once a week, and in a day care centre for older people. With few exceptions, caregivers reported negative experiences with bringing their relatives to a day care centre. One reason was the group size, with too many unknown people. Another reason was the lack of meaningful activities. One daughter (D63) reported that nothing happened; her mother did not do anything there and said it was “*boring to be there*”. Activities reported were a cup of coffee when they arrived and a small meal at noon.

The participants experienced day care centre services as inactive or passive services, without stimulating activities. One of the spouses (FS82a) said, *“He (my husband) felt he was put away and then he jumped off and came home”*. One of the male spouses explained that it was exhausting to motivate his wife to go out of the house, and that any activity outside their home involved a lot of planning and initiation. Despite experiencing limitations with the service, one of the spouses confirmed that he sent his wife to the day care centre anyway, because he needed some time for himself.

Some participants had experiences from short-term stays for their spouses in nursing homes, where all, except for one, had experienced this as ´a place for storage´. Both day care centre services and short-term stays in institutions created negative associations and most of the caregivers preferred to have their relatives at home.

Despite the negative experiences, the spouses stated that they would be prepared to facilitate joining activities outside their home, and accept health care services, if they knew that their wives or husbands would be satisfied when they returned back home. One of the wives wanted to highlight a positive experience she had; the bus driver who did motivate her husband to attend the day care centre once a week: *“The driver picks him up at nine o’clock and brings him back home again at three o’clock. He is amazing and this arrangement with this driver works well, I think”.* (FS82b).

### Lack of personalisation

Most participants stated that they were relatives of physically fit people with dementia. Some were even more physically fit than the caregivers: “*They have no services to suit my husband, he is physically fit, and I cannot manage to support him in activities he is interested in.”* (FS82c). Activities at the day care centres were not tailored to people’s physical functional levels. One of them gave an example of seated exercise classes despite the fact that her husband usually could walk long distances outdoors.

Most participants reported that their relatives would be able to cook meals and do several instrumental activities of daily living (I-ADL), with just a little support. Existing services, however, did not offer this kind of help. *“They can provide help in warming up ready meals; they can put it in the microwave oven. What they cannot do, is to help her (my wife) to turn the burner on when she is going to boil the potatoes.”* (MS75).

The participants felt that most of the health carers did not have any consideration towards the person’s unique identity and personality. One spouse (FS69) said: *“My husband is not ill - he has short term memory loss.”* As soon as the diagnosis of ‘dementia’ is given, focus seemed to be on the disease. The participants felt, like one of the daughters explained, that nobody was interested in “*the person behind the dementia*” (D63).

### Caregivers’ role is all-consuming

The participants reported that they were homebound because of their caring situation and they experienced this as a burden. This was particularly the case for the spouses who lived together with the person with dementia. They pointed out three challenges: an experience of being *the sole caregiver*, a *loss of social networks*, and a feeling that the increased burden of care resulted in *conflicting desires*.

### The sole caregiver

They explained that being the main person in their spouse’s, or parent’s, life gave them a lot of responsibility. They were often the only person that the person with dementia would receive any help from: “*He (my husband) does not allow others to come into our home. He trusts me and that I will look after him, for the rest of the life.”* (FS82c).

The participants explained that because of the large responsibility of being the main caregiver, they did not have time for doing other activities, either alone or together with their relatives. *“Yes, it is like fighting fires. We wish to do activities – nice things together with our moms, but we are caught in … .fighting fires.”*(D63).

### Loss of social networks

Participants who lived together with a person with dementia talked about their loss of social networks. Added to the challenges of supporting a relative with dementia was the social isolation that their situation created. One of the spouses put it like this: *“for me, the social aspect is the major problem.”* (MS75).

Some of the spouses expressed disappointment about previous friends and family members who did not visit them anymore “*… we do for example not have colleagues, persons at the same age; they do not exist in our life anymore. They have withdrawn.”* (MS75).

### Conflicting desires

Most of the participants expressed the feeling of being pulled in two directions. On one hand, they needed time to do things on their own; on the other hand, they wanted to have time to plan enjoyable activities for both of them. Most of them were unable to choose between activities they could do together or activities they could do alone: “*I would like to have some time for myself. Actually it would have been nice to do something together, but it is hard.”* (MS81).

#### Involvement and participation is necessary to improve services

According to the participants, the key to improving the health care services is to involve both users and caregivers in designing the services. More structured service systems and enhanced cooperation seemed to be vital. To improve the health care services, the caregivers suggested two factors: person centred services and individual support.

### Person centred services

To be able to support home-dwelling older people with dementia, the participants highlighted the importance of individualisation of the service, based on personal resources. Services should value the individual and focus on the skills that the person still has. To maintain activities of daily living, the health care services should focus on facilitating and supporting people to continue to perform these activities. As one of the wives said, her husband was able to do much more than health carers expected of him, with just a little support. One daughter (D63) confirmed this, while talking about her mother: *“If I prepare the food she is going to cook, she is able to cook dinner. If the task is simple, she can manage to do it.”*

The caregivers suggested that if the health care staff had known each individual, they could have brought people with similar interests together so that they could have enjoyed each other’s company.

### Individualised support

One daughter (D63) reported that she and her husband had supported her mother when shopping. *“She got the exercise while walking, she often met neighbours and the shopping activity as a whole was a positive experience* … w*hat if somebody from the home services in the municipality could have supported my mom in shopping.”*

The participants highlighted that it can be difficult to encourage people with dementia to participate in activities offered by the health carers. One of them pointed out the importance of involving staff that know the people with dementia when providing services: *“I think that if the same people came to their home, then she would have definitely been more confident and safe.”* (D63).

One of them (FS69) suggested that a companion could make access to activities easier: *“This is something I wish, that someone could have come to join him, like a companion, so he can go swimming, because he needs support.”*

The participants wanted to continue to be husbands, wives or daughters, rather than unpaid carers. *“Yes, energy that could have gone back to them. For example, if I have had time, I could manage to find concerts or other cultural activities that I can do together with them (my parents), but now I cannot manage to do this.”* (D63).

## Discussion

In the present study, we performed two focus groups with eight caregivers of older adults with a dementia diagnosis. With a few exceptions, we recruited caregivers with high levels of caregiver burden, shown as high scores for anxiety and depression and relative’s distress. Seven of them were caregivers for relatively physically fit persons, and they received limited help from the health care system. They all wanted to contribute to a better understanding of caregivers’ total situation, in order to improve health care services for people with dementia and their caregivers.

Overall, caregivers experienced a gap between current health care services and perceived needs, and they experienced services characterised by passivity and lack of individualisation. They wanted help or support to maintain performance in instrumental ADL functions, rather than services with compensational strategies (for example offering pre-prepared meals instead of supporting the person in the kitchen). This gap might explain the limited usage of health care services, as seen in the Norwegian study of resource use in this population [7]. Offers available for people with dementia were in generic settings for older people; people without similar needs or interests, possibly with lower physical abilities. Lack of personalised and differentiated health care services made them feel responsible for supporting their spouses or parents themselves. This feeling of lack of personalised and individualised services compounded the fear of perpetuating existing myths and stereotypes, like Rimmer et al. (2005) describes, which can be one reason why some caregivers hesitate or delay seeking help [6].

Participants in this study felt alone with the responsibility for their relatives, which created a great burden. They did not have any time left to do pleasant and enjoyable activities together with their spouses or parents. For the spouses, this situation made them homebound, with loss of social networks, which exacerbated their feelings of loneliness and pressure [6]. For the daughters, their involvement consisted of providing help instead of being daughters with time for social and pleasant activities.

Caregivers did feel that health carers focused mainly on the dementia diagnosis, exemplified through the statement “*My husband is not ill, he has short-term memory loss”*. All participants wanted health carers to show an interest for the person with dementia as an individual, in line with the recommendations for a person-centred approach and care [27]. According to our participants, future health care services for people with dementia should be person-centred, and health carers should support and enable people with dementia to use the personalised offers. Caregivers in this study had experiences from a very small number of health offers, which could indicate a lack of knowledge about available services. Information from health carers about available services may support informal caregivers in the help-seeking process, which also could increase access to and use of health care offers [12]. Supporting caregivers in this way may also contribute to a decreased perceived burden.

The caregivers in this study cared for people with dementia who were over 65 years of age, with most of them considering their relatives to be physically well-functioning. It could be that caregivers felt the day care services on offer were stigmatising, as they did not identify themselves with the target group for these offers. In line with an individualised approach, the caregivers highlighted the importance of considering the individual’s physical abilities when designing and delivering services. They do not want passive support. Offering people with dementia support in, for example, performing ADL activities in their own homes will require health carers to consider the person’s skills and abilities and involve them in line with his or her functional and activity levels. In accordance with Pinqart and Sørensen (2006), interventions should be tailored to the person’s functional level and needs [10]. One approach could be to regard the daily, regular routines already being a part of the person’s life, which could help both to delay functional decline for the person with dementia and support caregivers in a positive way. A person-centred approach, which values both people with dementia and those who care for them, and treats them all as individuals, will be a preventive strategy, which could delay the need for institutional care.

Our participants wanted to contribute to improvements in the existing services. They want to be involved when new services are developed, and they have serveral suggestions. One suggestion is to involve a support person, both to encourage participation in activities, and to provide support in transportation from the person’s home to the site where the activity is located. They also suggested that activities outside the home should be organised in small group settings, or through making sure that group members attending have similar interests. This is in line with others suggesting that services outside their homes need to be organised so they can be utilised [28]. The caregivers in this study highlighted that the content in all offers should be relevant for the people that attend. This will probably increase the motivation for participation for the person with dementia, and should also make the caregiver feel more positive about making the effort to access services.

It is likely that caregivers’ burden relates to their role as being the only support person, as well as the effect of the loss of a social network. The overall scores on the caregiver’s distress/HADS scores indicate that these caregivers already are in a high-risk group for psychiatric morbidity [20]. However, the caregiver’s burden may also be related to health care services, which do not meet their needs. Services that meet the needs as they have suggested, could improve the services, increase the use of these services, and ultimately support both caregivers and the person with dementia that live at home.

### Strength and weaknesses

We designed this study to gather the views of a small number of caregivers for people with dementia and wanted to explore their experiences in depth, in order to design new and better services. The findings are in line with work in other areas regarding the importance of person-centered care and services [27], adding validity to our research. We experienced that focus group interviews as the method, provided an opportunity for sharing difficult and emotional experiences as caregivers. The small number of participants is a limitation. We did, however, discuss the findings with additional caregivers to verify the conclusions we had drawn [25, 26]. The recruitment procedure could also be a limitation, where we recruited participants from a counselling service in the municipality and most of them participated already in conversational groups. The high scores on caregiver burden and symptoms of anxiety and depression could indicate that the recruited sample already had recognised the need for support due to their situation and we do not know how representative these participants are.

## Conclusion

We included a sample of caregivers of people with dementia that typically had many responsibilities and high caregiver burden. They highlighted that health care services did not meet their own needs, nor the needs of the person with dementia. They emphasised that services for people with dementia should give value to both the person with dementia and their caregivers through individualised and personalised approaches. Caregivers reported that services offered in the municipality had the diagnosis in focus rather than tailoring the services to the individuals’ physical function, interests and needs. Results from this study challenge researchers, clinicians, and health leaders in developing new services, or to improve current services, that can support caregivers and help people with dementia to stay active and healthy for as long as possible.

## Abbreviations

D: Daughter
FS: Female spouse MS Male spouse
HADS: the Hospital Anxiety and Depression Scale
RSS: the Relatives’ Stress Scale
